# A Mental Health–Informed Physical Activity Intervention for First Responders and Their Partners Delivered Using Facebook: Mixed Methods Pilot Study

**DOI:** 10.2196/23432

**Published:** 2021-04-22

**Authors:** Grace McKeon, Zachary Steel, Ruth Wells, Jill Newby, Dusan Hadzi-Pavlovic, Davy Vancampfort, Simon Rosenbaum

**Affiliations:** 1 School of Psychiatry University of New South Wales Sydney Australia; 2 St John of God Richmond Hospital St John of God Health Care North Richmond Australia; 3 School of Psychology University of New South Wales Sydney Australia; 4 Black Dog Institute Prince of Wales Hospital Sydney Australia; 5 Department of Rehabilitation Sciences KU Leuven Leuven Belgium; 6 University Psychiatric Center KU Leuven Leuven-Kortenberg Belgium

**Keywords:** physical activity, PTSD, social media, first responders, mental health, families, online, exercise

## Abstract

**Background:**

First responders (eg, police, firefighters, and paramedics) are at high risk of experiencing poor mental health. Physical activity interventions can help reduce symptoms and improve mental health in this group. More research is needed to evaluate accessible, low-cost methods of delivering programs. Social media may be a potential platform for delivering group-based physical activity interventions.

**Objective:**

This study aims to examine the feasibility and acceptability of delivering a mental health–informed physical activity program for first responders and their self-nominated support partners. This study also aims to assess the feasibility of applying a novel multiple time series design and to explore the impact of the intervention on mental health symptoms, sleep quality, quality of life, and physical activity levels.

**Methods:**

We co-designed a 10-week web-based physical activity program delivered via a private Facebook group. We provided education and motivation around different topics weekly (eg, goal setting, overcoming barriers to exercise, and reducing sedentary behavior) and provided participants with a Fitbit. A multiple time series design was applied to assess psychological distress levels, with participants acting as their own control before the intervention.

**Results:**

In total, 24 participants (12 first responders and 12 nominated support partners) were recruited, and 21 (88%) completed the postassessment questionnaires. High acceptability was observed in the qualitative interviews. Exploratory analyses revealed significant reductions in psychological distress during the intervention. Preintervention and postintervention analysis showed significant improvements in quality of life (*P*=.001; Cohen *d*=0.60); total depression, anxiety, and stress scores (*P*=.047; Cohen *d*=0.35); and minutes of walking (*P*=.04; Cohen *d*=0.55). Changes in perceived social support from family (*P*=.07; Cohen *d*=0.37), friends (*P*=.10; Cohen *d*=0.38), and sleep quality (*P*=.28; Cohen *d*=0.19) were not significant.

**Conclusions:**

The results provide preliminary support for the use of social media and a multiple time series design to deliver mental health–informed physical activity interventions for first responders and their support partners. Therefore, an adequately powered trial is required.

**Trial Registration:**

Australian New Zealand Clinical Trials Registry (ACTRN): 12618001267246; https://anzctr.org.au/Trial/Registration/TrialReview.aspx?ACTRN=12618001267246.

## Introduction

### Background

First responders, including police, firefighters, and paramedics, are regularly exposed to traumatic events. This repeated exposure puts them at an increased risk of experiencing psychological distress and poor mental health [1]. Overall, 1 in 3 first responders report having high or very high levels of psychological distress [2], whereas 1 in 10 first responders experience posttraumatic stress disorder (PTSD) [3]. Consequently, first responders are more than twice as likely to report having suicidal thoughts and are 3 times more likely to have a suicide plan than the general population [2].

First responders are also at an increased risk of poor physical health because of a number of occupational risk factors. Regular exposure to high-pressure situations, shift work, and physical inactivity contribute to high rates of cardiovascular disease and its risk factors, including hypertension and obesity [4]. Notwithstanding the physical demands of the role, obesity and hypertension remain significant problems among first responders [5-7]. The risk of physical health comorbidities is compounded in those with poor mental health [8,9]. For example, PTSD is a risk factor for obesity, diabetes, and metabolic syndrome [10-14]. Urgent efforts are needed to prevent and treat physical and mental health issues in this underserved population.

There is strong evidence showing that exercise and physical activity can help prevent and treat common mental disorders experienced by first responders (eg, depression, anxiety, and PTSD) while simultaneously improving physical health outcomes [15]. Physical activity has been shown to improve mood, reduce symptoms of depression and anxiety, improve sleep quality, and reduce alcohol dependence [15-18]. Improvements in mental health symptoms have also been seen in people with PTSD when delivered in addition to usual care [19].

Physical activity also has great potential as a preventative strategy. A review of existing studies suggests that regular physical activity is associated with a reduction in 17% of incident cases of depression [16]. This should be considered among populations at an increased risk of psychological injuries, such as first responders. Given that poor mental health is a risk factor for physical inactivity [20] and that physical activity levels decrease more steeply over time with increasing PTSD symptoms [21], early intervention and prevention strategies are critical for protecting long-term physical and mental health [8,9].

Despite the well-documented mental health and cardioprotective benefits of increased physical activity, there is limited research exploring ways to engage at-risk populations such as first responders in preventative or treatment-based exercise programs. Social media provides a unique opportunity to overcome barriers related to accessing care experienced by first responders, including issues related to stigma toward mental illness [22] and geographical barriers. Importantly, web-based platforms offer a cost-effective [23] and scalable opportunity to deliver mental health–informed physical activity interventions to the first responders living in regional and remote settings. Social media also promotes social connectedness, which is fundamental to exercise adherence and long-term behavioral change [24]. Facebook groups have previously been used effectively to deliver behavior change interventions in both the general population and in people with serious mental illness [25,26]. To our knowledge, no previous studies have tested Facebook as a means of increasing physical activity levels in first responders.

In developing programs to improve the physical and mental health of first responders, it is also important to consider the people who support them, including spouses, family members, and friends. Informal caregivers (eg, spouses and family members) are more likely to have depression, sleep problems, stress, and physical health conditions [27,28]. Carers, therefore, need access to support and programs to improve their mental health and well-being. Given the strong impact of social connections on health outcomes [29], particularly among dyads, the inclusion of nominated support partners is likely to be mutually beneficial.

### Objectives

This study aims to assess the feasibility, acceptability, and preliminary effectiveness of using a private Facebook group to deliver a physical activity intervention for first responders and their selected support partners. This study also aims to assess the feasibility of applying a novel interrupted time series design to determine whether this methodology would be a feasible substitute for a control group in an adequately powered trial. Exploratory analysis aimed to identify associated changes in mental health symptoms, sleep quality, quality of life, physical activity, and social support. We hypothesized that the program would be feasible, and participants would increase their physical activity with subsequent positive effects on mental health outcomes.

## Methods

### Design

**Figure 1:**
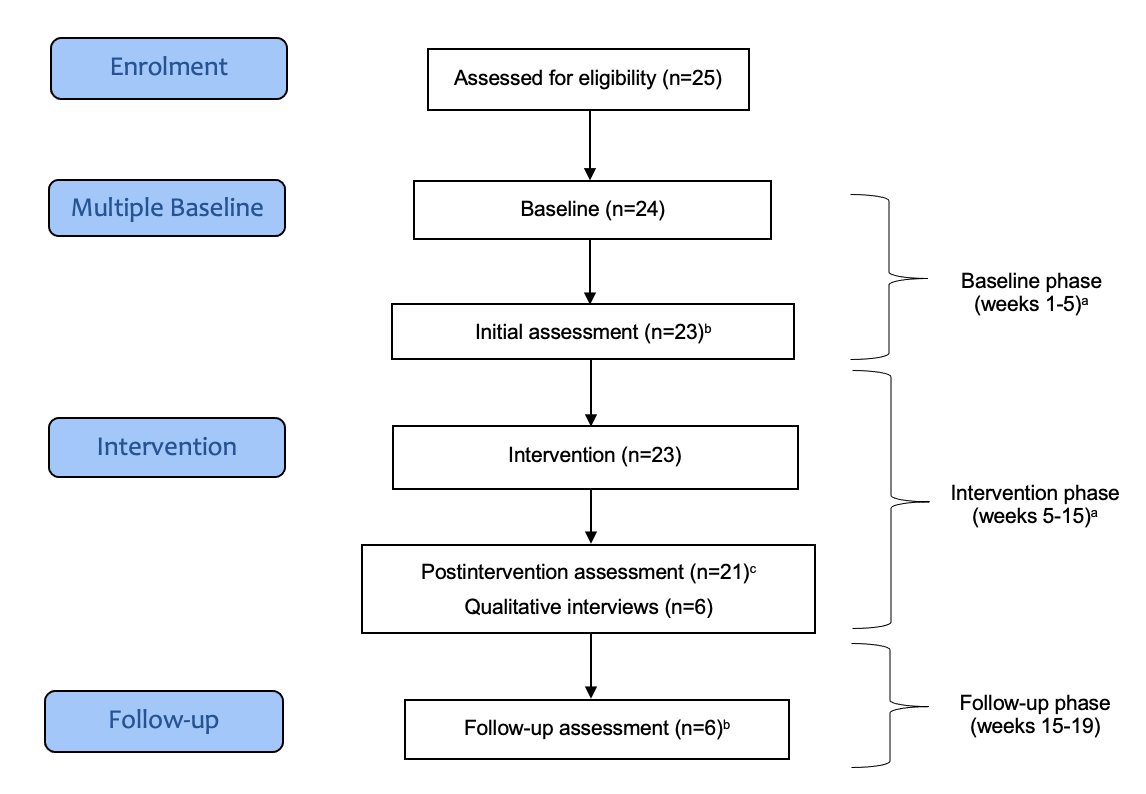
Flow of participants through the trial. ^a^Kessler-10 (assessed every 2 weeks); ^b^Depression Anxiety and Stress Scale-21, Pittsburgh Sleep Quality Index, Assessment of Quality of Life-6D, Simple Physical Activity Questionnaire, Posttraumatic Stress Disorder Checklist for Diagnostic and Statistical Manual of Mental Disorders, fifth edition (first responders only), and social support and exercise survey; ^c^Depression Anxiety and Stress Scale-21, Pittsburgh Sleep Quality Index, Assessment of Quality of Life-6D, Simple Physical Activity Questionnaire, Posttraumatic Stress Disorder Checklist for Diagnostic and Statistical Manual of Mental Disorders, fifth edition (first responders only), social support and exercise survey, and feasibility questionnaire.

An interrupted time series design was pilot tested. This methodology was used to detect whether the intervention had a significantly greater effect than the underlying secular trend [30,31]. Consecutive observations of the Kessler-10 (K10) every 2 weeks for a baseline period of 5 weeks were interrupted by the intervention to determine whether the series’ slope or level changed following the intervention. Participants, therefore, acted as their own control. This methodology was tested to inform the practicality of applying it to an adequately powered trial and replacing the need for a randomized controlled trial (RCT). A flow chart of the study design and assessment time points is shown in Figure 1.

This study was approved by the University of New South Wales Human Research Ethics Committee (HC180561) and prospectively registered (ACTRN12618001267246). All participants provided informed consent.

### Participants

Participants were recruited between August and September 2018 through *Behind the Seen*, a not-for-profit community initiative that aims to increase awareness and reduce stigma toward mental health issues faced by first responders and their families. The *Behind the Seen* facilitators posted the study advertisement on their Facebook page, which has a following of 25,000 people. Convenience sampling was used to recruit participants who met the following criteria: former or current first responders, aged between 18 years and 65 years, and who were currently physically inactive, which is defined as engaging in less than 150 minutes of moderate-to-vigorous physical activity (MVPA) per week [32] and was assessed using a physical activity vital sign questionnaire [33]. Participants also needed to be able to communicate in English and have internet access. Participants were eligible to participate if they answered *no* to all the questions in the Exercise Sports Science Australia Prescreening Tool [34].

First responders nominated support partners to participate in the program. This was defined as any person with close personal relationships with them, for example, partners, family members, caregivers, or friends. The support partner was required to be aged between 18 years and 65 years and be medically cleared to exercise. First responders or support partners who scored >30 in the K10 (indicative of very high levels of psychological distress) and who were not receiving treatment or whose medications had changed in the past 4 weeks were excluded and referred to a local health professional.

### Intervention

Participants were enrolled in a 10-week physical activity program delivered via a private Facebook group, which was set up for the purpose of the study. All participants commenced the program at the same time, which ran from October 2018 to January 2019. The facilitators of the group were exercise physiologists who provided education and facilitated discussions on different predetermined topics weekly, including goal setting, overcoming barriers, reducing sedentary behavior, and improving diet. A full description of the topics and example posts is provided in Table 1. All aspects of the program were codeveloped with the facilitators of the community organization (Behind the Seen), who have lived experience of both working as a first responder and living with PTSD. The facilitators posted 2-4 times per week and encouraged participants to post photos, ask questions, and share their journey on the Facebook group. The facilitators monitored the group daily from Monday to Friday and provided encouragement in the form of likes and comments in response to participants’ posts. The content and features of the program were co-designed with lived experience personnel and were based on behavior change techniques, including shaping knowledge, self-monitoring, and social support [35]. Content was posted weekly in the form of information, exercise demonstration videos, links to existing web-based resources, step count competitions, and discussion questions. Each participant was given a physical activity tracking device (Fitbit Flex 2) to monitor their activity levels. Participants were instructed on the different Fitbit functions, for example, how to track the intensity of their exercise and set goals using the phone app. Facilitators also set up group challenges using the Fitbit phone app, for example, the highest step count between first responders and support partners.

**Table 1:** Facebook group facilitator content.

Week	Topic	Content
1	Welcome	Participants were asked to introduce themselves (eg, occupation) and mention why they joined the group Instructions were provided on Fitbit activity trackers
2	Goal setting	How to write a SMART^a^ goal. For example, participants were encouraged to increase their step count from the last week by 5%-10% Participants were asked to write goals (short term, long term, and one with the support person) and post them on the Facebook group Benefits of self-monitoring and ways to do it (eg, Fitbit and training diaries)
3	Benefits of physical activity	Link between physical and mental health explained Physical and mental health benefits (eg, improved mood, sleep, and decreased anxiety and stress) Video links and factsheets provided
4	Barriers	Participants were asked to vote on their biggest barriers to getting active (eg, lack of time, low motivation, and low mood) Discuss strategies to overcome barriers and ask participants to share their suggestions and ideas
5	Support	Information provided on how to be a helpful support person (practical support, effective communication, and exercising together) Information provided on social support for increasing motivation
6	Sedentary behavior	Risks associated with sedentary behavior (eg, increased mortality risk) How to increase incidental activity, including ways to incorporate physical activity into everyday life Minimize time spent sitting and encourage breaking up long periods of sitting
7	Aerobic exercise	Australian guidelines (150 min of moderate-to-vigorous physical activity) Finding an exercise you enjoy
8	Resistance exercise	Australian guidelines (strength training at least two times per week) Exercise safety (eg, the importance of a warm-up) Videos of simple workouts (eg, squats and push-ups against a wall) The importance of progression and ways to do it (eg, using the FITT^b^ principle)
9	Healthy eating	The healthy eating pyramid Creating a healthy food environment (eg, shopping and cooking together and eating meals without distractions)
10	Review	How to maintain an exercise program Community programs discussed (eg, gyms and community runs) Review of goals Celebration of progress

^a^SMART: specific, measurable, achievable, realistic, and timely.

^b^FITT: frequency, intensity, time, and type.

### Data Collection

Data were collected from both the first responder and their support partner via web-based self-report questionnaires, Fitbit data, and one-on-one Skype interviews. Data from all self-report questionnaires were gathered through the MetricWire platform, a phone app.

### Measures of Feasibility and Acceptability

A manual calculation of the sum of posts, likes, comments, and views was performed to define Facebook group use, as has been similarly defined in previous research [36]. Feasibility was assessed based on participant retention, measured in 2 ways: first, the number of participants who remained in the group until program completion, and second, the number of participants who completed all of the postintervention assessments.

Acceptability was assessed postintervention using a 14-item feasibility and acceptability questionnaire developed for a private Facebook group [37]. Responses were answered on a 7-point Likert scale (strongly disagree to strongly agree). Acceptability was assessed using qualitative interviews. Participants were invited to participate in a 20-minute one-on-one semistructured interview via Skype following the intervention period. The interview covered topics including likes, dislikes, effectiveness, and recommendations for future iterations. The interviews were recorded and transcribed verbatim. Data were analyzed using thematic analysis to determine key themes identified by participants [38].

### Secondary Outcomes

#### Psychological Distress

The K10 self-report questionnaire was used to assess levels of psychological distress [39]. It consists of 10 items scored on a 5-point Likert scale, with total scores ranging from 10 to 50. Scores were grouped into 4 levels of psychological distress: low (scores of 10-15), moderate (scores of 16-21), high (scores of 22-29), and very high (scores of 30-50) [40]. The K10 has excellent psychometric properties [41], including high internal consistency (α=.93) [39] and discriminant validity [42].

#### Depression and Anxiety

The 21-item Depression Anxiety and Stress Scale (DASS-21) was used to assess the effects of the program on mental health symptoms [43]. Participants used the 4-point severity or frequency scale to rate the extent to which they had experienced each state *over the past week*. A total score and 3 separate subscales, each with 7 items, were calculated to identify severity ratings for depression, anxiety, and stress. Higher scores represented more severe symptoms. For the depression domain, scores of 0-4 were considered normal, 5-6 mild, 7-10 moderate, 11-13 severe, and >14 extremely severe. For anxiety, 0-3 was considered normal, 4-5 mild, 6-7 moderate, 8-9 severe, and >10 extremely severe. For stress, 0-7 was normal, 8-9 mild, 10-12 moderate, 13-16 severe, and >17 extremely severe. The psychometric properties of the DASS-21 have been comprehensively evaluated, and it has been found to be valid, consistent, and responsive to treatment [44,45].

#### Sleep Quality

The Pittsburgh Sleep Quality Index (PSQI) was used to assess participants’ quality and patterns of sleep in the past month [46]. This self-report questionnaire was assessed at baseline, postintervention, and follow-up. A total of 7 subscores were calculated, ranging from 0 to 3, to yield a global score ranging from 0 to 21. A sum of 5 or greater indicates a poor sleeper. Acceptable measures of internal homogeneity, consistency (test-retest reliability), and validity were obtained. The components of the PSQI were shown to have a high degree of internal consistency (α=.83) [46].

#### Quality of Life

The Assessment of Quality of Life-6D (AQoL-6D) scale was used to assess quality of life [47]. A total simple psychometric score for health-related quality of life and profile scores for the different dimensions were calculated. The 6 dimensions include independent living, relationships, mental health, coping, pain, and senses. Scores can range from 20 to 99, with lower values representing a better quality of life. The AQoL-6D questionnaire has achieved construct validity and provides a sensitive description of health-related quality of life [47].

#### PTSD Symptoms

The Posttraumatic Stress Disorder Checklist (PCL-5) in the *Diagnostic and Statistical Manual of Mental Disorders* (Fifth Edition) (*DSM-5*), is a 20-item self-report measure used to assess the *DSM-5* symptoms of PTSD [48]. Only the first responders were administered this questionnaire. Symptom severity scores range from 0 to 80, with a cutoff score of 33 indicating a provisional diagnosis of PTSD [49]. A decrease in scores of >10 points indicates a clinically significant change; >5 points indicates a reliable change. The PCL-5 is a psychometrically sound measure of *DSM-5* PTSD symptoms. It shows strong internal consistency (α=.94), test-retest reliability (*r*=0.82), and validity [48].

#### Social Support to Exercise

The social support and exercise survey was used to assess the level of support individuals making health behavior changes (exercise) felt they were receiving from family and friends [50]. Respondents rated the frequency with which family members and friends had done or said what was described in the item during the previous month on a 5-point scale, ranging from 1 (none) to 5 (very often). The survey was assessed at baseline, postintervention, and follow-up. The test-retest reliabilities of the factors were adequate, and the internal consistency reliabilities were high (α range .61-.91).

#### Minutes of Physical Activity

Physical activity levels were assessed subjectively using the Simple Physical Activity Questionnaire (SIMPAQ). The SIMPAQ is a 5-item clinical tool designed to assess physical activity among populations at high risk of sedentary behavior, with demonstrated validity and reliability among people with mental illness [51]. For the purpose of this study, the SIMPAQ was adapted to a web-based version using MetricWire. The SIMPAQ score was assessed at baseline, postintervention, and follow-up. The total time per week of walking, MVPA, and being sedentary was assessed.

### Procedure

Consecutive observations of the K10 were assessed every 2 weeks as part of the interrupted time series design. During the 5-week baseline period, the K10 was asked at 3-time points before the intervention. At the end of the baseline period (before intervention), the DASS-21, PSQI, AQoL-6D, PCL-5, social support to exercise, and SIMPAQ were assessed, as shown in Figure 1. During the intervention, the K10 continued to be assessed every 2 weeks, with a total of 5 time points during this period. At the end of the 10-week intervention, all outcomes were reassessed, in addition to the feasibility and acceptability questionnaire and the optional qualitative interviews. At the 4-week follow-up, all baseline questionnaires were reassessed.

### Statistical Analysis

In this study, we used two statistical approaches to test the changes during the intervention. First, we used a multiple baseline design to compare participants’ psychological distress levels with their own baseline (which acted as a control) using Mplus version 8. Second, we tested for differences between preintervention and postintervention outcomes on a battery of tests (open trial, no control) using SPSS version 24 (IBM Corporation).

#### Comparing Multiple Baselines With Intervention

A multiple baseline design was used to test for significant differences in participant trajectories between the baseline and during the intervention. A piecewise latent growth curve model was fitted in the Mplus base package using complex analysis. The model included a slope model for each participant’s K10 scores during the 3 baseline time points (baseline slope) and during the 5 intervention time points 4 to 8 (intervention slope). In latent growth curve models, each participant’s repeated time points were modeled as a latent variable of slope for each participant and a mean slope across the group. That is, a multilevel model was designed in which each participant’s time series is nested within each individual, which is nested within the overall group. Random effects were used to allow each participant to have their own intercept and slope. An assumption of linear modeling is that the observations between participants will be independent. However, partners in this study were likely to share characteristics and were thus nonindependent; clustering by partnerships was included to account for the nonindependence of results within pairs. A piecewise model can model 2 separate slopes for each participant within a time series (between baseline and intervention). Model constraints were used to test for significant differences between the slope estimates between baseline and intervention.

#### Pre-Post Intervention Tests

A multilevel modeling approach was used to test repeated measures for each individual, accounting for pairs (first responder and support partner). That is, while accounting for clustering within pairs, mixed models were run to test whether participants showed significant changes in outcome measures. Directional hypotheses were used (one-tailed tests), and Cohen *d* effect sizes were calculated. All analyses for the pre-post tests were conducted on SPSS version 24.

## Results

### Participant Demographics

In total, 24 participants (13/24, 54% female, 12/24, 50% first responders and 12/24, 50% support partners) were recruited for the study. Table 2 presents the demographic characteristics of the participants. The relationship between most participant-support partner pairs was life partners (10/12, 83%). Half of the first responders were former or current firefighters, 33% (4/12) were paramedics, and 17% (2/12) were police officers. Most of the total sample (17/24, 71%) used Facebook for >3 hours per week before the intervention. The flow of participants throughout the study is shown in Figure 1.

**Table 2:** Baseline demographics.

Characteristic		First responder (n=12)	Partner (n=12)	Total (N=24)
Age (years), mean (SD)		48.1 (11.12)	44.8 (12.13)	46.5 (11.5)
Sex (male), n (%)		10 (83)	1 (8)	11 (46)
Smoker, n (%)		2 (17)	2 (17)	4 (17)
**Marital status, n (%)**				
	Married	7 (58)	7 (58)	14 (58)
	Single	2 (17)	1 (8)	3 (13)
	Other or prefer not to say	3 (25)	4 (34)	7 (29)
**Education, n (%)**				
	High school	2 (17)	3 (25)	5 (21)
	Diploma or certificate	8 (66)	2 (17)	10 (42)
	Bachelor or postgraduate degree	2 (17)	7 (58)	9 (37)
**Occupation, n (%)**				
	Police	2 (17)	2 (17)	N/A^a^
	Fire	6 (50)	N/A	N/A
	Paramedic	4 (33)	1 (8)	N/A
	Nonfirst responder	N/A	9 (75)	N/A
**Work status, n (%)**				
	Current serving	7 (58)	N/A	N/A
	Retired or medically discharged	5 (42)	N/A	N/A
**Relationship with the first responder, n (%)**				
	Life partner	N/A	10 (84)	N/A
	Friend	N/A	1 (8)	N/A
	Family member	N/A	1 (8)	N/A
**Facebook use per week, n (%)**				
	3+ hours	11 (92)	6 (50)	17 (71)
	1-3 hours	N/A	4 (33)	4 (17)
	<1 hour	1 (8)	2 (17)	3 (12)

^a^N/A: not applicable.

### Feasibility and Acceptability

#### Retention

From a total of 24 participants, 21 (88%) completed the postintervention assessment questionnaires and 22 (92%) completed the program. One participant dropped out during the baseline period, before the intervention. Another participant dropped out during week 8 for unknown reasons. Participants completed 98% of the multiple time series design K10 assessments, which occurred at 8 time points across the baseline and intervention period. Only a minority (6/24, 25%) completed the 4-week postintervention follow-up assessment.

#### Engagement

Over the 10-week study period, there was a total of 544 likes and comments from 23 participants. There were a total of 76 individual posts, with 50 posts (66%) coming from participants and 26 posts (34%) from facilitators (Table 3). The total post views of the first responder, support partner, and facilitator-initiated responses were high, with a mean of 18.4 (SD 3.5) views per post across the 10 weeks, indicating an average view rate of 83% (Table 3). The highest response rate was a total of 26 comments and 5 likes from 12 participants and was in response to a post on goal setting. Poll responses were not included in the sum of likes and comments but received the highest interaction. Participants’ posts included screenshots of their daily step counts, community exercise programs, personal barriers to getting active, and screenshots of sleep patterns as recorded by the Fitbit. Participant-initiated posts declined across the intervention, with the lowest being week 9, which focused on diet.

**Table 3:** Engagement with Facebook posts initiated by first responders, partners, and facilitators.

Week, topic, and position		Number of posts	Post views, mean (SD)	Likes, mean (SD)	Comments, mean (SD)
**1, Welcome**					
	First responders	13	16.5 (1.8)	3.3 (1.6)	3.5 (2.1)
	Partners	4	18 (0.8)	4.8 (3.0)	3.8 (1.5)
	Facilitators	4	11.5 (1.3)	3.8 (1.9)	9.6 (7.1)
**2, Goal setting**					
	First responders	6	18.8 (1.7)	5.2 (1.5)	8 (3.7)
	Partners	0	N/A^a^	N/A	N/A
	Facilitators	4	19.5 (1)	6 (1.8)	6.8 (12.8)
**3, Benefits of physical activity**					
	First responders	1	23 (0)	5 (0)	2 (0)
	Partners	1	21 (0)	6 (0)	4 (0)
	Facilitators	2	21 (1.4)	4.1 (2.1)	1.5 (2.1)
**4, Barriers**					
	First responders	4	23.2 (0.9)	5.5 (1.3)	3.8 (1.3)
	Partners	1	23 (0)	1 (0)	7 (0)
	Facilitators	3	20.7 (1.5)	5 (5.3)	5.3 (3.8)
**5, Support**					
	First responders	2	18 (1.4)	2 (0)	1 (1.4)
	Partners	1	21 (0)	4 (0)	0 (0)
	Facilitators	2	19 (1.4)	7.5 (4.9)	6 (5.7)
**6, Sedentary behavior**					
	First responders	4	20.6 (1.7)	5.5 (1.7)	4 (2.1)
	Partners	2	21 (4.2)	6 (1.4)	4 (4.2)
	Facilitators	3	19 (1.4)	2.5 (3.5)	4 (4.2)
**7, Aerobic exercise**					
	First responders	4	20.3 (1.0)	6.5 (3.7)	1.8 (1.0)
	Partners	3	20.7 (3.2)	1.7 (0.6)	5 (3.0)
	Facilitators	2	19 (1.4)	2 (0)	1.5 (0.7)
**8, Resistance exercise**					
	First responders	3	22.7 (1.2)	5 (2.6)	1.7 (1.2)
	Partners	0	N/A	N/A	N/A
	Facilitators	2	20.5 (2.1)	4 (2.8)	3.5 (3.5)
**9, Healthy eating**					
	First responders	0	N/A	N/A	N/A
	Partners	0	N/A	N/A	N/A
	Facilitators	2	20 (0)	3 (1.4)	4 (0)
**10, Review**					
	First responders	1	17 (0)	0 (0)	5 (0)
	Partners	0	N/A	N/A	N/A
	Facilitators	2	20.5 (4.2)	9 (0.7)	6 (1.4)

^a^N/A: not applicable.

#### Fitbit Compliance

From a total of 24 participants, 22 participants (92%) downloaded the Fitbit app and activated their device. Once this setup had occurred, they were worn on 89% of the days during the 10-week intervention period and on 48% of the days during the 4-week follow-up period. From the 22 participants, 19 participants (86%) wore Fitbits >80% of the days during the 10-week program, and 21 (95%) wore them >50% of the days.

#### Acceptability

**Figure 2:**
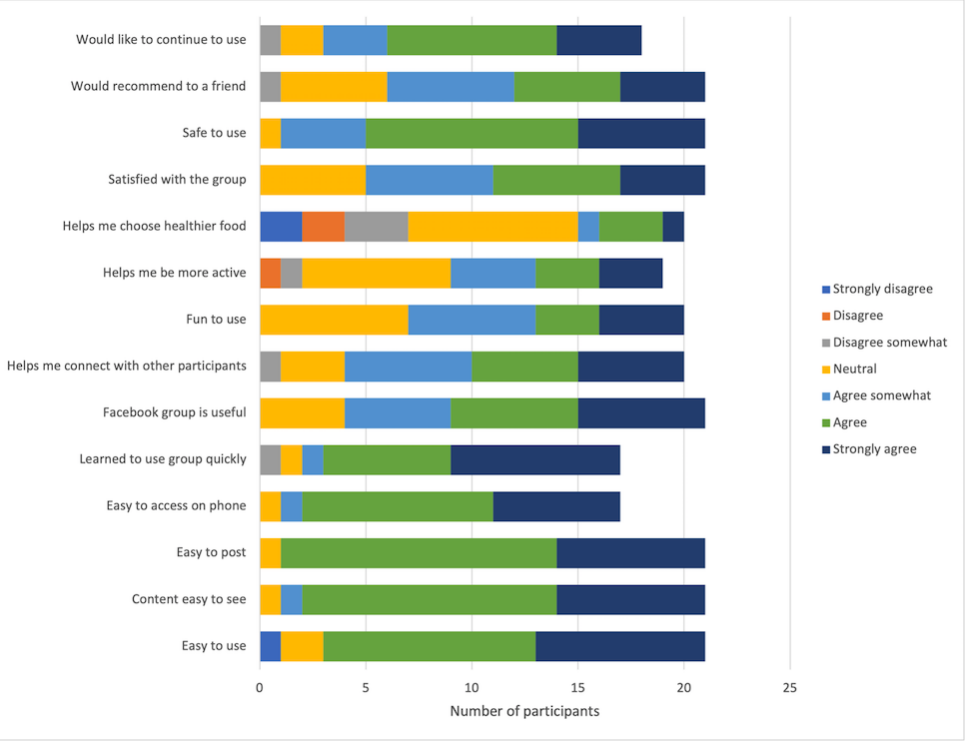
Participant responses to the feasibility and acceptability questionnaire.

Figure 2 summarizes the participants’ responses to a feasibility and acceptability questionnaire. On average, both participants and their support partners appraised the program positively. Mean scores across the 14 questions were 5.5 out of 7, meaning participants somewhat agreed or strongly agreed that the group was feasible and acceptable. Mean scores ranged from 3.79 to 7.0. Most of the participants agreed or strongly agreed that the Facebook group was easy to use (18/21, 86%) and useful (17/21, 81%); importantly, 95% (20/21) said it was safe to use. There was some discrepancy in whether the group helped participants be more active or eat healthier food; 7 participants disagreed to some degree that the program helped them eat healthier, and 2 participants disagreed that it helped them be more active. In addition, 5 first responders and 1 support partner participated in the optional qualitative interviews. All 6 participants experienced high or very high psychological distress levels at baseline, and engagement levels with the page varied between them. A total of 5 themes were identified from the data: (1) the role of physical activity and exercise for mental and physical health, (2) the contributing role of individual intervention components (eg, Fitbit and Facebook page), (3) the significance of social support, (4) identity and camaraderie associated with being a first responder, and (5) opportunities for future work and recommendations.

Selected participant quotes relating to key themes are shown in Table 4. Participants identified that regular exercise improved their mental health by helping them better manage their symptoms. A subtheme emerged for physical activity acting as a catalyst for functional recovery, for example, facilitating participation in social activities. Participants reported that the use of Fitbit increased awareness of physical activity levels. Some participants reported that they would like to have seen more interaction on the Facebook page, whereas others appreciated the lack of pressure to contribute. Overall, the social support received from partners and other members of the group was well accepted and highly valued, and participants also reported forming friendships with other members of the group outside of the program. The camaraderie of being a first responder emerged as a theme, with participants appreciating that others in a similar position to themselves were participating and supporting each other.

**Table 4:** Participant quotes from the qualitative interviews.

Themes and subthemes			Examples of supporting quotations
**Benefits of physical activity**			
	Mental health	“Definitely my consistency increased. And if I look at my data on my phone, or when I sync it, I can see that when I regularly exercise and don’t miss a class and stuff, I certainly am a lot better mentally.” “...but I was talking to my psychiatrist yesterday and he was saying definitely increasing my exercise has helped me with my sleep because we’ve looked at my data on Fitbit and three months ago I wasn’t sleeping as good as what I am now.” “Like, I know when I do my exercise,...I’m not thinking about my problems, I’m not thinking about any crappy job I’ve gone to, it’s a form of mindfulness almost. It’s a form of I’m being right present what I’m doing because I don’t want to get injured, so I’m trying to do it right, so I am really concentrating on me and the task at hand which then takes the focus off your depression, or PTSD, or anxiety.” “And I just know hand in hand for me that when I don’t exercise, my mental health deteriorates. When I exercise regularly and consistently, my mental health stays stable.”	
	Catalyst for functional recovery	“When my son says, ‘Hey dad, you want to go tobogganing after school,’ I’m like, ‘Yes, let’s go.’	
	Management tool	“It’s made me go out more. I find me going outside more, even if I don’t feel like exercising. So, that for me, that’s a big improvement, and I feel much more—it doesn’t stop the posttraumatic stress. It doesn’t stop me having a meltdown every time someone mentions something. But I don’t stay stuck in the meltdowns now.”	
**Role of intervention components and study design**			
	Fitbit	“It was sitting there, I’m conscious that it’s there because I can see it. And so it’s like, oh yes, I need to be active. Does that make sense?...It was an actual talisman to trigger you to do things.” “And it did, I was more mindful of being more physical because I was wearing it. It was a reminder.” “But definitely having the Fitbit and I appreciate that so much because that’s made a world of difference to my life insofar as I sync it every night. And, you know, some nights I haven’t got to 10,000 steps, so I get up and I go and walk until I finished it.” “You’ve created this new—‘conscience’ is a good word, I think. You’ve created this new conscience within me because you’ve given me something that is a tangible and physical reminder of a need for change.”	
	Facebook group	“You guys did great. You could share as much as you wanted or you could just observe as much as you wanted. It was, and there was no pressure. I knew that if I was having a rough couple of days and I didn’t say anything on there it wasn’t taken negatively.” “Look, I think the threads and the general chats were good, absolutely. I just think it would be good if people just opened up a little bit more. Like, I was reserved and I didn’t say much at all, to be honest. And in hindsight, I probably should have. But then you don’t want to bombard.” “It’s all positive. I think anything like this there are no negatives. I’d like to say thank you for involving us in it. It’s been great. I’ve had probably the worst year of my life this year and it’s been good to have this, the yoga, and I’ve been getting back into music and I’m just finding it’s been good having those balanced pillars and this has been part of it.” “It encourages people. I’ve made contact with a few people out of it. I keep in contact with XXX from XXXX...So yes, the Facebook site’s good.” “It’s awareness. I know that, I can say oh yes, I know I’ve got to eat better, I know I’ve got to do more exercise. As soon as you’ve got 30 people that are looking at what you’re doing like, I’m like oh shit, they can see what I’m doing on my Fitbit. So having a certain level of responsibility, almost. But it was very light, like, you guys didn’t care at all what we did, I don’t think. You were just, we’ll see what happens. And that was really comforting, knowing that there was zero pressure.” “Guess I was probably looking for more because the problem I had was being motivated. I even had the same trouble speaking to my own psychologist, psychiatrist. I want to do it but I really struggle to motivate to do it, to exercise.”	
	Questionnaires	“So I gather they are the bread and butter of gathering information at any given point in time. But yes, they are a real pain in the arse to answer.”	
**Significance of social support**			
	Support partners	“So having the Fitbit was like, it was a reminder to her that she needs to take care of herself just as much as I do and that we are a team. So it was like, how many steps did you have today. She’s like oh my gosh, I only got 2,000.” “XXX and I bouncing it off each other at the end of the day I’m like okay, how many steps did you do, that sort of—not in a competitive way but it was another avenue to be able to go okay, how was your day? How active were you? That sort of thing. So we would compare a little bit like that.”	
	Support group	“It was a really good group where there was supportive people and I really connected with XXX, and he connected with XX as well because he’s all about yoga and peer support stuff. So we made a friendship. It’s pretty awesome that way. We’re lucky.” “...you have created community, you have created a conversation.”	
**Identity**			
	Camaraderie of being a first responder	“So that, knowing that there are other people out there doing a similar thing, involved in a similar study, and knowing, being aware that whatever data comes from this and future studies is going to help people. That gives me some encouragement and joy because that’s in my nature; I wouldn’t have spent so long as a paramedic.” “But knowing that there’s a group of people out there doing the same sort of thing—I’m tribal and it’s kind of, I know ambulance and police and ADF, we are tribal by nature so there is a real comfort in being part of another tribe.”	
**Opportunities for future work and recommendations**			
	Peer support	“So yes, you guys just—you’re doing amazing things. Keep up the good work and I’m hopping on-board. I’ll help in any way that I can.”	
	Recommendations	“...and I guess the other thing is trying to get people outside...and a big thing for me walking was taking a photo - stopping to notice, and it became really mindful...For me, I realised with the posttraumatic stress, if I have an anxiety attack and I stop, and I just take a photo of something around me, of nature around me, it drops the anxiety attack out. So, it’s adding that little bit of mindfulness to the walking.” “I would have liked to have seen a little bit more interaction on the Facebook page. I didn’t think there was a huge amount of interaction on the Facebook page either. Particularly towards the end, I felt like I was commenting, and I was commenting too much, because no one else was really doing that.” “And even just these phone calls from you guys before you start because then that you actually feel it in your voice and even over a phone and get a feeling for a person, then just an email or just a comment on a Facebook page.”	

### Secondary Outcomes

#### Psychological Distress

Examination of the individual K10 means showed that participant trajectories of change did not form a straight line, meaning that in some weeks, participant levels of psychological distress would increase, whereas in others weeks, they would decrease, that is, there were nonlinear trends across time. A quadratic trend was therefore used to test for a change in the direction of the slope. The model showed an excellent fit (*X*^2^_1_=27.5; *P*=.38; root mean square error of approximation=0.05; comparative fit index=0.992; Tucker-Lewis index=0.991; standardized root mean square residual=0.086). The baseline slope was not significant (b=−0.09; *P*=.77). The intervention slope showed a significant decrease across the intervention period (b=−1.067; *P*=.003; ie, despite changes in direction, the overall K10 scores decreased during the intervention period). There was a significant quadratic trend over the intervention (b=0.148; *P*=.046), meaning that the change trajectories showed a change in direction during the intervention period. Model constraints showed a trend toward differences between the baseline and intervention slopes, but this was not significant (b=0.977; *P*=.09). Although there was no significant change at baseline and a significant change during the intervention, there was no significant difference between the 2 slopes.

### Preintervention and Postintervention Outcomes

The results for all other secondary outcomes at baseline and postintervention are shown in Table 5. Significant improvements in the total DASS-21 scores (*P=*.047; Cohen *d*=0.35) and AQoL-6D (*P*=.001; Cohen *d*=0.60) were observed. Changes in sleep quality (*P*=.28; Cohen *d*=0.19) and social support from family (*P*=.07; Cohen *d*=0.37) and friends (*P*=.43; Cohen *d*=0.02) were not significant. Changes in mean PCL-5 were also not significant (*P*=.10; Cohen *d*=0.38); however, 36% (4/11) of the first responders’ PCL-5 scores decreased by >10 points, indicating a clinically significant change in that group, and 55% (6/11) of the first responders’ PCL-5 scores decreased by >5 points, indicating a reliable change. Self-reported average minutes of walking per day showed a significant effect on time from baseline to postassessment (*P*=.04; Cohen *d*=0.55). No statistically significant changes in self-reported levels of sedentary behavior (*P*=.18; Cohen *d*=0.22) or MVPA (*P*=.11; Cohen *d*=0.34) were found.

**Table 5:** Preanalysis and postanalysis of secondary outcomes.

Variable and position			Baseline, mean (SD)		Postintervention, mean (SD)		Time					Position^a^					Time×position		
							*F* test^b^		*P* value		*F* test^b^			*P* value		*F* test^b^			*P* value
**DASS^c^-21 total**																			
	First responder	23.4 (11.2)		20.5 (13.3)		3.09		*.047* ^d^		1.54			.23		0.35			.56	
	Partner	19.4 (14)		13.5 (11.4)		N/A^e^		N/A		N/A			N/A		N/A			N/A	
**DASS-21 depression**																			
	First responder	8.5 (3.9)		7.4 (6.1)		2.22		.08		4.40			.*048*		0.13			.72	
	Partner	5.6 (4.1)		3.7 (3.3)		N/A		N/A		N/A			N/A		N/A			N/A	
**DASS-21 anxiety**																			
	First responder	5.3 (2.9)		4.4 (3.7)		6.02		*.01*		0.54			.47		1.28			.27	
	Partner	4.9 (4.4)		2.6 (3.5)		N/A		N/A		N/A			N/A		N/A			N/A	
**DASS-21 stress**																			
	First responder	9.3 (4.8)		8.2 (4.6)		1.94 (1)		.09		0.00			.99		0.11 (1)			.75	
	Partner	8.5 (6)		6.7 (5.6)		N/A		N/A		N/A			N/A		N/A			N/A	
**PSQI^f^**																			
	First responder	12.7 (2.6)		12.1 (4.6)		0.34		.28		0.42			.53		3.18			.09	
	Partner	9.3(5.5)		8.1 (5.5)		N/A		N/A		N/A			N/A		N/A			N/A	
**AQoL-6D^g^**																			
	First responder	48 (7.9)		43.6 (9.5)		13.64		*.001*		2.64			.12		0.02			.89	
	Partner	44.1 (7.9)		38.4 (8.1)		N/A		N/A		N/A			N/A		N/A			N/A	
**Mental health**																			
	First responder	12.9 (2.6)		10.8 (2.6)		15.85		*.001*		1.33			.26		0.03			.86	
	Partner	11.4 (3.9)		9.4 (3.7)		N/A		N/A		N/A			N/A		N/A			N/A	
**Senses**																			
	First responder	5.3 (1.2)		5.6 (1.6)		1.07		.16		0.74			.40		0.27			.61	
	Partner	4.9 (1.3)		5.0 (1.2)		N/A		N/A		N/A			N/A		N/A			N/A	
**Relationships**																			
	First responder	6.7 (1.5)		5.6 (1.2)		8.46		*.004*		0.88			.36		0.75			.40	
	Partner	6.0 (1.6)		5.4 (1.2)		N/A		N/A		N/A			N/A		N/A			N/A	
**Independent living**																			
	First responder	8.2 (2.4)		7.3 (2.2)		6.43		*.01*		4.586			*.04*		0.143			.709	
	Partner	6.9 (1.9)		5.6 (1.2)		N/A		N/A		N/A			N/A		N/A			N/A	
**Pain**																			
	First responder	6.2 (2.5)		5.5 (2.1)		2.85		.05		0.04			.99		0.01			.89	
	Partner	6.4 (2.6)		5.8 (1.9)		N/A		N/A		N/A			N/A		N/A			N/A	
**Coping**																			
	First responder	10.2 (2.5)		8.6 (1.8)		18.31		*<.001*		4.09			.06		0.17			.68	
	Partner	8.6 (1.8)		7.2 (1.8)		N/A		N/A		N/A			N/A		N/A			N/A	
**Family social support**																			
	First responder	20.6 (7.6)		19.8 (5.3)		2.50		.07		0.56			.46		4.25			.05	
	Partner	18.3 (4.6)		24.9 (10.9)		N/A		N/A		N/A			N/A		N/A			N/A	
**Friend social support**																			
	First responder	19.5 (8.4)		18 (4.8)		0.03		.43		0.00			.99		1.63			.22	
	Partner	21.1 (9)		22.7 (8.7)		N/A		N/A		N/A			N/A		N/A			N/A	
**PCL-5^h^**																			
	First responder	39.3 (18.8)		32.3 (21.1)		1.93		.10		N/A			N/A		N/A			N/A	
**Sedentary time, hours/day**																			
	First responder	9.1 (3.5)		9.3 (3.5)		0.92		.18		0.08			.78		1.45			.25	
	Partner	10.4 (4.4)		8.7 (2.6)		N/A		N/A		N/A			N/A		N/A			N/A	
**MVPA^i^, minutes/week**																			
	First responder	72.27 (114.8)		130.91 (125.3)		1.67		.11		0.03			.86		0.42			.53	
	Partner	84.0 (127.3)		103.5 (129.7)		N/A		N/A		N/A			N/A		N/A			N/A	
**Walking, minutes/day**																			
	First responder	56.82 (56.5)		87.73 (67.2)		3.616		*.04*		0.55			.47		0.10			.75	
	Partner	68.0 (72.7)		111.3 (82.1)		N/A		N/A		N/A			N/A		N/A			N/A	

^a^Position indicates first responders or support partners.

^b^Degrees of freedom=1 for each interaction.

^c^DASS: Depression Anxiety and Stress Scale.

^d^Italics indicates statistical significance.

^e^N/A: not applicable.

^f^PSQI: Pittsburgh Sleep Quality Index.

^g^AQoL-6D: Assessment of Quality of Life. For AQoL-6D and its subscales, lower scores indicated better health.

^h^PCL-5: Posttraumatic Stress Disorder Checklist for Diagnostic and Statistical Manual of Mental Disorders (Fifth Edition).

^i^MPVA: moderate-to-vigorous physical activity.

## Discussion

### Principal Findings

To the best of our knowledge, this is the first study to assess the feasibility, acceptability, and preliminary effectiveness of using a private Facebook group to deliver a physical activity intervention for first responders and their support partners. Current efforts to implement physical activity programs for populations at risk of poor mental health are often hindered in their scalability and sustainability by factors such as the high cost and lack of accessibility. Our study demonstrated that social media is a feasible and acceptable platform for delivering a mental health–informed physical activity intervention, and exploratory analysis of secondary outcomes appears promising.

Our study’s retention rates were high, with 92% (22/24) of participants remaining in the group and completing the 10-week program and 88% (21/24) completing the postassessment questionnaires. This is in line with the current literature, where compared with traditional web-based interventions, web-based social networks typically achieve high levels of user engagement and retention [52]. A systematic review of studies that used Facebook to deliver physical activity interventions found a comparable attrition rate of 9% [53]. The multiple time series design also proved to be feasible, with 98% of possible K10 assessment time points being completed by the participants. Satisfaction ratings were high, with participants agreeing that the Facebook page was safe and easy to use. On average, both first responders and their support partners stated that they would continue using the group and recommend the program to a friend. Having service end users involved from the initial development of the program is likely to have contributed to the high feasibility and acceptability observed in our pilot study.

There was strong compliance with wearing the Fitbits with regard to the percentage of days worn (89% of days) and high satisfaction reported in the interviews with using the wearable devices for activity tracking. This is consistent with previous studies on people with severe mental illness [54]. The use of existing low-cost and widely available wearable technologies that enable participants to see one another’s achievements and challenge each other may be particularly useful in partner support interventions targeting lifestyle change.

A decline in participant-initiated posts was observed over the 10-week intervention period; however, total views per post remained, with posts being viewed on average by 83% of participants. Research has shown that web-based interventions with a social media component help to sustain engagement [52]. Clinicians and researchers delivering web-based interventions should, however, be aware that it is difficult to determine engagement with web-based interventions [55]. Engagement does not necessarily predict effectiveness, and there is evidence showing that those lurking in online support groups may benefit to the same extent or even more than regular posters [56,57]. A potential strategy to address the decline in posts may be to have volunteers from previous iterations act as peer support members in future groups. Druss et al [58] have shown that peer support is a promising strategy for helping people change their lifestyle behaviors. In their study, 400 participants with serious mental illnesses were provided 12 sessions of a chronic disease self-management course led by mental health peers with chronic conditions, and they found statistically significant improvements in mental and physical components of quality of life and in recovery assessment scores. Peers could act as powerful role models for changing behaviors and may increase retention by relating to participants’ challenges and offering personal advice [59].

Given that this was a pilot study, interpreting the secondary outcome data should be done with caution and treated as exploratory, not hypothesis testing. Although the sample size is small, it is consistent with the intent of pilot studies as an initial step in exploring the feasibility and potential benefits of a novel intervention [60]. A total of 61% (14/23) of participants experienced high or very high levels of psychological distress at baseline. As expected, there was no significant change in distress levels during the baseline period. A significant decrease in psychological distress was seen across the repeated time points during the intervention; however, the interaction between the baseline and intervention slopes did not reach significance. We would anticipate that a larger sample size would provide sufficient power to observe significant interactions.

Significant improvements were observed in the quality of life and total DASS-21 scores. Unexpectedly, no changes in perceived social support to exercise were observed. Similarly, an RCT using Facebook to assess changes in perceived social support among university students also found no significant difference in perceived social support following a web-based intervention [25]. It should also be noted that the social support to exercise questionnaire used in this study did not differentiate between perceived support received from the other participants in the group, their partner, or the facilitators. Therefore, it remains unclear whether a web-based group-based program is effective for increasing perceived social support.

Changes in sleep quality and PTSD symptoms were not statistically significant in this small sample. A significant change in self-reported minutes of walking per week was observed, but not in sedentary time or MVPA. A possible explanation could be the large group SD, which is typical of self-reported physical activity [61]. In addition, our inclusion criteria did not exclude support partners who were sufficiently active at baseline. In a larger-scale trial, these secondary outcomes could be pooled across iterations, clustered by cohorts, to test for statistical significance with a larger sample size.

Importantly, this pilot study focused on a major public health problem given the toll that providing care takes on people’s physical and mental health [62]. The benefits in mental health outcomes experienced by the support partners warrant extending lifestyle interventions to include spouses and family members. Meta-analytic evidence from 21 RCTs (involving 1589 participants) has shown that both support groups and group-based face-to-face psycho-educational interventions are effective for caregivers of patients with severe mental illnesses [63]. However, few studies have addressed lifestyle behaviors among support partners [64,65]. In line with the literature [62], the support partners were experiencing high levels of psychological distress at baseline, with 55% (6/11) of the support partners experiencing at least moderate levels of psychological distress and 36% (4/11) of the support partners experiencing high or very high levels of distress. There was no significant difference in trajectories between the first responders and support partners in any of the outcomes.

### Limitations and Future Research

Some limitations of this pilot study include the generalizability and inadequate statistical power. Our sample was small and involved only self-selected participants. In addition, the lack of age and gender diversity may limit the generalizability of these findings to other samples or broader characteristics of first responders, such as those with severe mental illness or those who are not active on social media. It is also difficult to dismantle the intervention components (eg, Fitbit and Facebook group) to determine which component has the greatest effect. Although there was a lack of a control group and a small sample size, the novel time series design proved to be feasible. A time series design should be considered when designing lifestyle interventions when RCTs may not be realistic or warranted; this design has been applied to a full-scale study of the program described here [66].

Another limitation was the insufficient follow-up data because of high attrition between postintervention and follow-up assessments. Therefore, it is inconclusive whether the short-term benefits obtained through this type of intervention would be maintained over the long term. Web-based programs are uniquely placed as content can be delivered more regularly than face-to-face interventions and accessed in the participants’ own time. A short 10-week program may also be an important initial step in fostering autonomous motivation and self-efficacy to engage in further physical activity programs. Future research should examine whether web-based interventions have the ability to maintain positive behavior changes over the medium and long term. Future researchers should consider the views of posts as an important measure of passive engagement. Future studies should consider the cofacilitation of content delivery by peer support workers and the use of other Facebook group features, including video calls, to further enhance the participant experience.

### Conclusions

Delivering web-based interventions to increase physical activity among first responders and their support partners presents a potentially useful implementation strategy because of its low cost and ability to reach a large number of people. The results of our pilot study show that using Facebook to deliver a physical activity program and using a multiple time series design are feasible. Exploratory analysis showed significant improvements in mental health symptoms and quality of life. Given these results, a larger-scale trial is warranted, and future iterations should build on the strengths and weaknesses of this pilot study.

## Abbreviations

AQoL-6D: Assessment of Quality of Life-6D
DASS-21: Depression Anxiety and Stress Scale
DSM-5: Diagnostic and Statistical Manual of Mental Disorders (Fifth Edition)
K10: Kessler-10
MVPA: moderate-to-vigorous physical activity
PCL-5: Posttraumatic Stress Disorder Checklist for Diagnostic and Statistical Manual of Mental Disorders (Fifth Edition)
PSQI: Pittsburgh Sleep Quality Index
PTSD: posttraumatic stress disorder
RCT: randomized controlled trial
SIMPAQ: Simple Physical Activity Questionnaire
